# Challenges in Clinical Training for Nursing Students during COVID-19: Examining Its Effects on Nurses' Job Satisfaction

**DOI:** 10.1155/2024/7865540

**Published:** 2024-11-22

**Authors:** María-de-los-Ángeles Merino-Godoy, Emilia Teixeira da Costa, Marianela Gómez Salas, Alba Pavón Lara, Nicolás Carretero Bernal, Beatriz Macías Domínguez, Francisco-Javier Gago-Valiente

**Affiliations:** ^1^Nursing Department, Faculty of Nursing, University of Huelva, Huelva 21007, Spain; ^2^Nursing Department, Health School, University of Algarve, Faro 8000, Portugal; ^3^Health Sciences Research Unit, Nursing School of Coimbra, Coimbra 3000, Portugal; ^4^Residencia de Mayores Jesús de Nazaret, Gibraleón 21500, Spain; ^5^Complejo Asistencial de Ávila, Valladolid 47007, Spain; ^6^Hospital Universitario Miguel Servet, Zaragoza 50004, Spain; ^7^Centro de Investigación FABIS, Hospital Juan Ramón Jiménez, Huelva 21007, Spain; ^8^Center for Research in Contemporary Thought and Innovation for Social Development (COIDESO), University of Huelva, Huelva 21007, Spain

## Abstract

**Introduction:**

Nursing education involves a robust blend of theory and hands-on practice, crucial for cultivating the intricate abilities required to safely progress from being a student to becoming a proficient nursing professional. This training process was disrupted by the COVID-19 pandemic when the imposition of lockdowns compelled the transition of classes from in-person to online formats.

**Aim:**

This study aimed to assess the challenges in clinical training for nursing students during the COVID-19 pandemic, specifically examining how reductions in hands-on clinical practice have impacted their job satisfaction upon entering the workforce.

**Methods:**

It was an exploratory, descriptive, and cross-sectional study, using the Font Roja Questionnaire on job satisfaction as an instrument for data collection. The population was made up of Spanish nurses who graduated in 2020, 2021, and 2022.

**Results:**

The sample consisted of 390 nurses, 81.5% female, averaging 24.35 years old, with 76% having missed at least one month of clinical practice during their training. We found significant levels of dissatisfaction with job pressure and professional competence (52.3% and 40.8%, respectively). Statistically significant differences were found between gender, job pressure, year of graduation, and professional competence.

**Conclusion:**

The loss of clinical practice periods, a vital element in nursing education, has influenced the early careers of these nurses, particularly affecting certain aspects of their job satisfaction such as job pressure and professional competence.

## 1. Introduction

In Spain, after many changes in the nursing education and practice, including the nomination of practitioners in 1857, nurses in 1915, and health technical assistants in 1952, it was just in 2007 when the implementation of the Degree in Nursing was established [1]. This included a profound change in the training structure of these professionals, namely, the considerable increase in credits required in theoretical-practical hours [2]. Through this blend of theoretical knowledge and its practical application, which include all hands-on classes taught at the university itself and care practices in clinical contexts (hospitals and health centres, among others), the aim is to achieve a level of development of complex skills, necessary for a safe transition from nursing student to nursing professional [2].

In recent years, we experienced a serious global health emergency, the COVID-19 pandemic [3]. It was at the beginning of March 2020 when the World Health Organization (WHO) declared the problem of this virus as a pandemic and, in the month of April, more than two million people tested positive for the coronavirus, causing the hospitalization of many of them due to multiple complications associated with this type of pneumonia [4].

This situation has had repercussions on the health of many groups of workers, but especially on nurses, which are one of the most important groups of staff in our health system. This situation arose because nurses were tasked with providing care in environments that lacked sufficient resources for the emerging needs, amidst confusion and chaos caused by the unfamiliar circumstances encountered [3]. In addition, nursing care requires close contact with patients, leading to exposure to biohazards and a high probability of infection [5].

Since the beginning of the pandemic, immediate measures were implemented for a more effective protection of the population, which changed many aspects of our lives, as was the case of the adaptation of many jobs to teleworking, in the social sphere, confinement changed freedom of mobility for strict restrictions, and in the educational sphere, classes went from being face-to-face to online [6, 7].

This is why another group severely impacted by the pandemic was students, especially those with an advanced level of specialization such as university students. It all started with a long queue of online classes and a multitude of works that sought to replace activities that, previously, would never have been considered in any other way than face-to-face. For nursing students, the practical learning was replaced by online classes in which, through virtual platforms, illustrative videos, and forums, they were expected to learn (without practicing in a simulated or real context) important techniques and instrumental procedures that, in the future, should undoubtedly be carried out on and with patients [8, 9].

It should be noted that in the context of nursing professionals' training, the pandemic generated much controversy because within the international context, certain universities banned nursing internships, but in many others, 4th year nursing students were recruited to work in healthcare facilities. This was done without the availability of sufficient resources and protective measures, so that the students who worked, many of them voluntarily, were infected in the same way as the health professionals themselves [10]. According to previous research, those with the highest mental health impact from a pandemic are frontline professionals in the healthcare system and students nearing the end of their degree, as they undertake large-scale clinical placements [11].

Despite the possibility offered at some universities for students to work, there were nursing students who have graduated without completing some important periods of practical learning. This may have had short-term effects such as irritability, anxiety, stress, and psychological discomfort [11] and it is possible that there may be medium- and long-term consequences for all students of these graduating classes in their entry into the world of work, given that practical and clinical preparation are a fundamental pillar in the training of future nurses, providing the development of skills and abilities essential for the profession [12]. The International Council of Nurses [13] recognized that these changes in clinical practice may have had an impact on the quality of the education of nurses qualified during this period and consequently on the quality of the resulting nursing working force. This could affect, among other issues, the level of job satisfaction of these new nurses.

Locke [14] argues that satisfaction is a pleasant or positive emotional state resulting from a subjective appreciation of the work experience and that it should be considered primarily as a product of performance, the result of the effectiveness of the action, evaluated by the individual. This view leads us to consider the possibility that nurses who were trained during the pandemic, because their training had some weak points, feel less competent and less effective in their work and that this may affect their level of satisfaction. It should be added that satisfaction is also considered an important component of the quality of care, particularly of the relationship established, in this case, between nurse and patient [15, 16]. In response to these considerations, we implemented an exploratory, descriptive, cross-sectional study targeting graduates from the years 2020 to 2022, employing a nonprobabilistic sampling approach. This method enabled swift engagement with a varied group of participants who began their nursing careers amid a global health crisis.

## 2. Purpose

This study aimed to assess the challenges in clinical training for nursing students during the COVID-19 pandemic, specifically examining how reductions in hands-on clinical practice have impacted their job satisfaction upon entering the workforce.

## 3. Materials and Methods

### 3.1. Study Design and Participants

Our study employed an exploratory, descriptive, cross-sectional design using nonprobabilistic sampling methods. We focused on a group of nurses who graduated during the years 2020, 2021, and 2022. This group was specifically chosen because all members completed their education during the pandemic, providing a unified characteristic central to our research objectives. We selected a nonprobabilistic sampling strategy because it enabled us to rapidly and effectively reach a broadly dispersed demographic across various regions. This approach was particularly advantageous for our exploratory study, as it allowed us to capture a diverse range of insights during the final stages of the pandemic, despite the remaining constraints and challenges.

The process of gathering data was conducted through the utilization of Google Forms, which were disseminated across various social media platforms, including Facebook, Instagram, and WhatsApp, between 11 February and 10 April 2023. We highlight the publications in forums related to nursing, in health dissemination accounts, and a free search of the target population in social networks through keywords referring to the inclusion criteria.

In selecting the most appropriate social media platforms for disseminating our questionnaire, we focused on Facebook, Instagram, and WhatsApp due to their widespread usage among healthcare professionals, as supported by recent studies indicating these platforms' popularity within the nursing community [17]. These platforms were deemed particularly effective for reaching a broad demographic of nursing graduates from 2020 to 2022.

To further target our audience, we identified forums and accounts that are actively engaged in discussion about nursing and healthcare. Selection was based on factors such as the active user base, the relevance of discussion to recent nursing graduates, and the geographic reach that aligns with our study population. Forums were chosen for their high engagement rates and their focus on professional development and pandemic-related healthcare challenges.

For the free search component, keywords were carefully selected to resonate with the experiences and challenges of recent graduates. These included terms such as “newly graduated nurse,” “pandemic nursing challenges,” and “nursing career during COVID-19.” This keyword strategy was designed to maximize exposure to our survey across these platforms, ensuring that we reached individuals directly impacted by the pandemic's influence on nursing education and entry into the profession.

The initial sample consisted of 400 participants. Participants who had over 20% of their data missing were not included in the study, amounting to a total of 10 individuals. Ultimately, the sample consisted of 390 participants who met the following specific criteria: (a) nurses who have completed their degree training during the COVID-19 pandemic, graduated in 2020, 2021, or 2022; (b) who have already entered the labor market as nurses; (c); who reside in Spain and possess the ability to comprehend and read Spanish; (d) who willingly agreed to take part in the study upon reviewing and understanding the terms of the free and informed consent. To ensure a broad representation, we included participants from a variety of Spain's autonomous regions, each with its unique healthcare settings and policies. This variability is crucial as it influences the professional experiences and training challenges faced by nurses.

Based on the nature of the data collection method, we identified factors for exclusion: limited digital literacy, inability to access the Internet or lack of social media presence, and failure to respond to at least 80% of the total questions on the form. Furthermore, our recruitment strategy was designed to promote inclusivity, extensively using nursing forums and social media networks, thus mitigating potential selection bias and enhancing the generalizability of our findings.

Data collection encompassed various regions and universities because the regulatory framework for nursing education and practice is uniformly dictated by national standards within each country and supplemented by directives across the European Union. This framework ensures that clinical training and required competencies are consistently implemented, which is essential for professional accreditation and practice.

The research was carried out in strict adherence to the ethical guidelines outlined in the Declaration of Helsinki. Approval for the study protocol was obtained from the Provincial Research Ethics Committee of Huelva (internal code: 0797-M1-23 | protocol code: AMG-COV-2021-0213). Prior to data collection, all participants were thoroughly briefed about the study's procedures and aims, and their informed consent was secured.

### 3.2. Measures

Nurses completed a brief *ad hoc* sociodemographic questionnaire, providing information about their age, gender, social status, country region where the degree was carried out, clinical practice lost due to COVID-19, graduation year, employment situation, and if they made clinical practices where they currently work. Specifically, by quantifying the amount of clinical practice lost during their training due to the pandemic, we are directly assessing the challenges that these disruptions posed to their preparedness and transition into professional nursing roles. Secondly, participants were asked to respond to the Font Roja scale [18] in its extended version [19] which allows measuring the degree of job satisfaction that these nurses have. This decision was driven by the scale's comprehensive approach to measure multiple facets of job satisfaction, including job-related stress and professional competence, which are critical in understanding the impacts of pandemic-induced disruptions on new nurses. The extended version offers additional items that capture nuanced aspects of the nursing experience under these unique conditions, ensuring a thorough evaluation of factors that influence job satisfaction. Furthermore, this version has been validated and shows high reliability [18, 19], making it highly suitable for assessing the target population's adaptation to professional roles during a period of unprecedented challenges. Thus, its use not only supports our investigation into the specific impacts of the pandemic but also enhances the depth and validity of our findings regarding the professional well-being of newly graduated nurses.

This tool (Table 1) presents 26 items divided into 10 different factors: job satisfaction (4 items), job-related stress (5 items), professional competence (3 items), job pressure (2 items), career advancement (3 items), interpersonal relationship with superiors (2 items), interpersonal relationship with colleagues (1 item), extrinsic status characteristics (2 items), job monotony (2 items), and satisfaction with the physical work environment (2 items). The response to this instrument is done on a Likert scale, measured from 1 (highly disagree) to 5 (highly agree). The score obtained in each dimension is the sum of all the questions that make it up divided by the total number of questions. The result ranges from 1 to 5 points, with 3 being the cut-off point. Thus, nurses with a score of 3 or more will be considered “satisfied,” while those with a score of less than 3 will be considered “dissatisfied.” The global scale of Cronbach's *α* is 0.791 [19], and in the present study, we obtained 0.826.

### 3.3. Statistical Analysis

Statistical analysis was conducted using SPSS® software, version 28. Initially, we assessed the distribution of our data for normality using the Kolmogorov–Smirnov test, which yielded a *p* value of 0.200. This result indicated a normal distribution, thereby justifying the use of parametric tests for further analyses.

In the processing of the data, we conducted both descriptive and exploratory analyses. For qualitative variables, we reported absolute (*n*) and relative (%) frequencies. For quantitative variables, we calculated measures of central tendency and variability, including the mean, standard deviation (SD), minimum (min.), maximum (max.), and range, which provided a comprehensive overview of the data spread and central points.

To compare group means, the Student's *t*-test was utilized for two group comparisons, and the simple analysis of variance (ANOVA) was employed for multiple group comparisons. To account for multiple comparisons and minimize Type I errors, Bonferroni's adjustment was incorporated into the ANOVA tests. This rigorous adjustment ensures that the findings remain robust even under the scrutiny of multiple comparisons.

Statistical significance was established at a *p* value less than 0.05. This threshold was chosen to balance the risk of Type I and Type II errors, ensuring that results deemed significant are both statistically robust and relevant to our research objectives.

These statistical methods were selected to align with the specific characteristics of our dataset and the exploratory nature of our study, aiming to accurately identify and interpret the potential impacts of the pandemic on the professional experiences of newly graduated nurses.

## 4. Results

### 4.1. Sociodemographic and Academic Characterization

The sample consisted of 390 nurses. The distribution followed by the population based on gender was 81.5% women (*n* = 317) and 18.5% men (*n* = 72). The average age of the population was 24 years. Most of the population was single as a marital status (92%).

Regarding the location where the nurses did their studies, the sample comes from different Spanish communities such as 35.6% from Andalusia (*n* = 139), Valencia 15.6% (*n* = 61), Madrid 8.2% (*n* = 32), Catalonia 5.9% (*n* = 23), and Murcia with 4.9% (*n* = 19), among other communities. The distribution by graduation year was 19.7% (*n* = 77) in 2020, 36.7% (*n* = 141) in 2021, and 43.8% (*n* = 171) in 2022. A significant majority of nurses have carried out internships during the pandemic period (78.7%); however, in many of these situations, the study plan did not follow its normal course. Due to all the limitations imposed by COVID-19, the missed internship for 24% (*n* = 94) was 1 month or less, 1 month to 3 months for 56.2% (*n* = 219), for 13.6% (*n* = 53), from 3 months to 6 months, and losses greater than 6 months for 6.2% (*n* = 24). The distribution of the loss of clinical practices based on the year of graduation allows us to conclude, as can be seen in Table 1, that the nurses who lost the greatest number of clinical practices were those who graduated in 2022. Finally, 84.6% of the participants are currently working as nurses, and in relation to whether they have carried out clinical practices in the services where they currently work, 65.8% (*n* = 256) have carried out them and 34,4% (*n* = 134) have not.

The nurses surveyed who were not currently working, had recently worked, and were asked to refer to their last place of work when assessing satisfaction. The results relating to sociodemographic and academic characteristics are presented in Table 2.

### 4.2. Job Satisfaction

**Figure 1:**
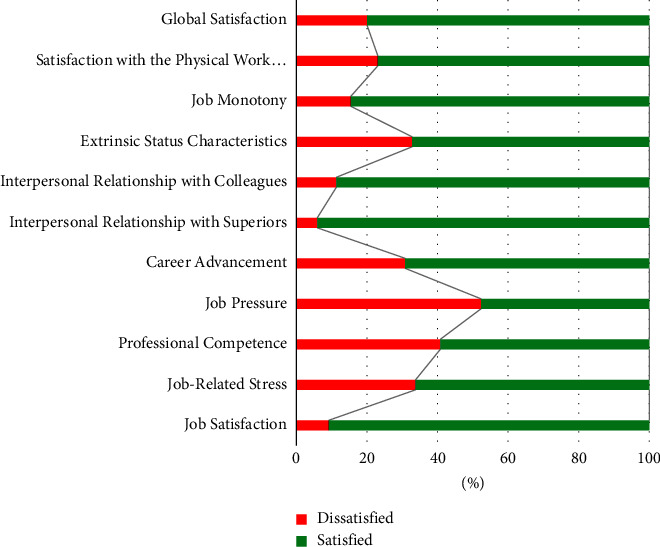
The dispersion of satisfaction based on scale factors (*N* = 390).

The scale factors were analyzed according to the authors' suggestions [19], classifying the nurses as satisfied (scoring 3–5) or dissatisfied (scoring 1-2). We found that for the scale's total score, most respondents (80%) considered themselves satisfied with their profession. However, when the assessment is made separately for each of the 10 factors that make up the questionnaire, the levels of satisfaction vary, as can be seen in Figure 1. We would like to highlight the most relevant dissatisfaction values as the job pressure factor, related to the grade of overburden due to the work position with 52.3%, the professional competence factor related to the grade of coincidence between professional training and work position with 40.8% and the job-related stress factor, associated to the grade of stress induced by the profession of the worker, and reflected by fatigue and the grade of responsibility perceived with 33.8%. The factors with the highest levels of satisfaction are, respectively, interpersonal relationship with superiors (94.1%), general job satisfaction (90.8%), and interpersonal relationship with colleagues (88.7%).

Following data analysis, relations between different variables were clarified. In a first examination, each of the academic variables was assessed in relation to the total score on the scale (26–130) to take advantage of the widest range of values; we found an average value of 87.95 (SD = 12.07) for the total instrument (min.47 and max.120). As can be seen in Table 3, no significant differences were found for these parameters. Nevertheless, we feel it is important to emphasize that, although not with statistically significant values, women, professionals who finished their training in 2020, those who did not practice during the pandemic, those who missed more than 6 months of clinical practice during their training, and those who did not have practices in the service where they currently work, are the ones with the lowest average job satisfaction values.

Considering the factors with the lowest satisfaction scores (Figure 1), we then proceeded to analyze them according to gender and academic variables. At this point, we used the factor scores as suggested by the authors [20, 21] from 1 to 5, considering that a score of 1 to 2 translates dissatisfaction and 3 to 5, satisfaction.

Significant statistical differences were observed in terms of gender concerning job pressure and professional competence. It was noted that females reported reduced satisfaction levels, particularly regarding the degree of stress associated with their job roles (job pressure) and the alignment between their professional training and their job positions (professional competence). In addition, the analysis revealed significant statistical differences in professional competence based on the year of graduation, with the year 2022 marking the lowest levels of satisfaction. We would also like to point out that although there were no significant differences, nurses who had lost more than 6 months of clinical practice had the lowest values for job pressure, professional competence, and job-related stress. The results can be seen in Table 4.

## 5. Discussion

The primary objective of this research was to evaluate the impact of the COVID-19 pandemic on the clinical training of nursing students and to explore how this has affected their professional satisfaction upon entering the workforce. Amid the pandemic, healthcare systems were overwhelmed, placing unprecedented pressures on newly graduated nurses. These graduates faced significant reductions in hands-on clinical practice due to lockdowns and safety measures, varying significantly by university, regional location, and timing of pandemic waves. Despite the maintenance of the required total credit hours, the shift from practical to theoretical online learning posed considerable challenges. Nursing, being a predominantly practical profession, relies heavily on clinical experiences that are difficult to replicate through online education. In our research, most of the population was female (81.5%), aligning with the traditional caregiving nurse role associated with women. This pattern is in line with the demographics of the nursing profession in Spain, where most professionals are women. In 2021, according to data from the National Institute of Statistics, 84.16% of nursing professionals in Spain were females, such as the composition of our study [22].

We found that of the 390 nurses who graduated during the pandemic, 76% missed at least one month of clinical practice during their training, with those who graduated in 2022 missing out on the most practical learning opportunities (44.1%). This considerable reduction in hands-on clinical experience is significant because such training is crucial for building the skills and confidence necessary to function effectively in clinical settings. The absence of this essential component due to pandemic restrictions undoubtedly posed a substantial challenge for these graduates, impacting their readiness and self-assuredness as they transitioned into their professional roles. Other authors have also found considerable changes at this level of learning in nursing education during the pandemic [23]. Because this element stands as a cornerstone in nursing education, the circumstances prevalent during the pandemic years significantly impacted the professionals who were trained during this period, undeniably shaping their entry into the workforce. Numerous research endeavors have emphasized the difficulties encountered by recently qualified nurses, including heightened stress, fear, and anxiety [23, 24]. In addition, they reported feeling ill-equipped to handle the new job requirements [25] and often experienced inadequate support due to limited staffing and resources, along with a lack of structured induction programs such as shadowing periods [26].

This research recognizes professional satisfaction as an important component of the healthcare quality and can even be recognized as one of its indicators [15, 16]. In this study, most of the participants (80%) reported being generally satisfied with their profession, scoring an average of 87.95. Some authors [27] classify this level of satisfaction as moderate. However, when we examined the individual factors within the questionnaire, we noticed that job pressure, professional competence, and job-related stress were the aspects that had the highest number of dissatisfied professionals. These findings are consistent with those of other research studies [27].

On the other hand, this study verifies that men have a higher overall job satisfaction than women, respectively, 90.53 and 87.37 (although no statistically significant differences are verified). In relation to this finding, other authors have also verified greater satisfaction in men [28] but in the same way without statistically significant differences [29]. However, when we analyzed the factors that make up the Font Roja scale, we did find significant differences with regard to gender, job pressure, and professional competence. In the nursing field, the observed higher levels of job satisfaction, better handling of job pressure, and greater perceived competence among men compared to women may be influenced by several factors, often rooted in societal norms, professional dynamics, and individual expectations [30].

This study also scrutinized the relationship between the year of graduation from nursing programs and subsequent job satisfaction levels. The analysis revealed variations in job satisfaction across different graduation years. Specifically, it was observed that nurses who obtained their degrees in 2020 exhibited lower job satisfaction compared to their counterparts from other years. We can attribute this variation to the 2020 cohort's curtailed practical training, a consequence of the pandemic's disruptive impact on educational programs. While the literature on this specific topic is scant, the referenced study from the AMN Healthcare [31] corroborates the trend of diminished job satisfaction among nurses with less experience, a group inclusive of recent graduates, underscoring the need for further investigation into this critical issue.

Upon examining the relation between graduation year and scale factors, it becomes apparent that the year 2022 is characterized by diminished satisfaction levels concerning professional competence. This observation is particularly significant, considering that the same period experienced the most substantial reduction in clinical practice opportunities due to the pandemic. Such a trend may elucidate the perceived mismatch between the theoretical education received and the practical demands of the professional roles undertaken. This pattern underscores a sophisticated comprehension of how hands-on clinical experience, job competence, and job satisfaction are interconnected, especially within the nursing sector. Contemporary studies corroborate that practical clinical training is a pivotal element, greatly enhancing both the professional capability and the contentment of nursing personnel. The emphasis on training underscores the critical nature of direct, experiential learning in fostering the necessary competencies and confidence in nurses, not only during their initial educational phase but also as an ongoing aspect of their professional development [32, 33].

In addition, we would like to point out that in our analysis, certain trends in job satisfaction emerged among specific subgroups, which, though not statistically significant, warrant further discussion to understand their potential implications. Notably, women, recent graduates from 2020, and those who either missed more than 6 months of clinical internships or did not perform internships during the pandemic reported lower levels of job satisfaction. In addition, individuals who did not undertake internships in their current places of work also reflected similar dissatisfaction. These patterns suggest that the disruptions caused by the pandemic might have had an adverse effect on these groups. Potential factors contributing to this dissatisfaction could include the lack of hands-on clinical experience crucial for building confidence and competence in nursing practice. Furthermore, the misalignment between the environments where training was received and where they are currently employed could exacerbate feelings of unpreparedness and ineffectiveness. Similar conclusions have been drawn by other researchers [32, 34, 35].

## 6. Conclusions

The investigation encompassed a cohort of 390 nursing professionals, predominantly females, with an average age around 24 years. A considerable number of these individuals experienced a disruption in their clinical training, missing out on a month or more of essential hands-on experience. The findings highlighted a discernible level of discontent particularly in relation to aspects such as professional competence and the stress associated with their roles. The study also pointed out that these sentiments of dissatisfaction varied notably when examined through the lenses of gender, the year they completed their education, and the extent of clinical practice they were unable to undertake. Yet, it is noteworthy that a vast majority, 80%, still felt overall satisfaction with their nursing career, despite these challenges.

The pandemic's disruption of clinical training was a key focus of this study. It underscored how missing out on this vital aspect of nursing education profoundly affected the early career stages of these nurses, particularly those who completed their education in 2022. When comparing male and female nurses, men reported slightly higher job satisfaction, although the difference was not statistically significant. The analysis indicated that the degree of job satisfaction showed variations relating to the graduation year, though these differences did not reach statistical significance. Notably, nurses who completed their education in 2020 exhibited lower levels of overall satisfaction compared to their peers from subsequent years. This pattern is likely connected to the reduced clinical practice hours they experienced. Furthermore, the broader context of global uncertainty and the specific challenges within the healthcare sector during their transition from education to practice could have further influenced their satisfaction levels. This combination of reduced practical training and the turbulent healthcare environment at the time of their graduation may have compounded the challenges faced by these nurses.

In summary, from analyzing the data from this study, we can conclude that the COVID-19 pandemic's interruption of clinical training had a significant effect on nurses' job satisfaction, especially in areas such as job pressure and professional competence. These findings emphasize the vital role of hands-on clinical training in preparing nurses, not just in terms of skills, but also in ensuring their job satisfaction and overall readiness for the profession.

## 7. Recommendations

The conclusions of this investigation highlight the profound repercussions of the COVID-19 pandemic on the clinical training and job satisfaction experienced by nursing graduates. These findings reveal crucial impediments that have adversely affected their professional competence and escalated job-related stress. Our study not only corroborates earlier research that has documented the acute challenges confronted by healthcare professionals during global health crises, but it also expands our comprehension by specifically quantifying the impacts on nurses who have recently graduated. In response to these findings, several strategic recommendations have been proposed to enhance the transition from nursing education to professional practice, particularly during challenging times such as a pandemic.

1. Enhanced psychological support systems: Observing the relatively high levels of job satisfaction amidst significant adversities underscores the importance of reinforcing this resilience through targeted support mechanisms. We advocate for the integration of counseling services and stress management workshops within healthcare organizations. Such initiatives would better equip novice nurses to manage the stress associated with their roles effectively.
2. Strengthened collaboration between academia and healthcare facilities: It is imperative to fortify the collaboration between educational institutions and healthcare facilities. This partnership should ensure that practical training is not only uninterrupted but also mirrors the current challenges prevalent in healthcare environments. By enriching clinical training modules and simulations that mimic real-world scenarios, particularly those involving infection control and emergency management, we can adequately prepare nurses to meet the demands of their professional duties.
3. Specialized short courses or certifications: Introducing specialized courses can further equip graduate nurses with critical skills. Courses on managing critically ill patients, emergency response, and health disaster management would be invaluable in boosting nurses' confidence and competence, thereby enhancing their job satisfaction.
4. Policy initiatives for continuous education and training: We urge policymakers to allocate funding for continuous professional development and to establish adaptive policies that ensure educational programs are capable of responding to unforeseen circumstances. Implementing regular assessment and feedback mechanisms to evaluate the effectiveness of these educational and support programs is crucial. These mechanisms will facilitate timely adjustments and ensure that the educational needs of nursing students are met efficiently and effectively.

In conclusion, by adopting these recommendations, healthcare and educational institutions can greatly enhance the preparedness and job satisfaction of nursing professionals. While the COVID-19 pandemic has concluded, the strategies developed in response to it hold valuable lessons for managing other types of crises, such as natural disasters, wars, and public health emergencies. Implementing these measures can bolster the resilience and capacity of healthcare systems, facilitating smoother transitions for nurses from educational settings to practical roles in times of crisis. Such efforts are vital for upholding high standards of care and creating a supportive environment for future generations of nursing professionals. These insights are critical not only for current challenges but also for equipping our healthcare systems to respond to future global health crises effectively.

## 8. Limitations

This study is subject to several limitations that must be considered when interpreting its findings. Firstly, the cross-sectional design restricts our ability to trace the longitudinal impacts of disrupted clinical training on nursing practice. While providing a snapshot of the effects at a specific point, this design limits our ability to infer causality or track changes over time, potentially affecting the validity of our findings and complicating their extrapolation to different contexts or over time. Longitudinal studies are required to capture these dynamics comprehensively.

Secondly, evaluating nurses with varying lengths of professional experience simultaneously may introduce biases related to different career stages. Despite implementing statistical controls for age, level of experience, and specific interruptions in training, this approach might not completely neutralize the effects of varied professional maturity on the adaptation to professional roles. This heterogeneity in responses could influence the internal validity of our study, suggesting that the findings may not accurately represent any single group of nurses.

In addition, the use of a convenience sample presents a further limitation, potentially leading to selection bias. Participants who are more accessible or willing to participate may not be representative of the broader nurse population, thereby limiting the generalizability of our results. The selection bias inherent in convenience sampling necessitates a cautious interpretation of the findings, particularly when considering their application to all nursing graduates affected by the pandemic.

Given these limitations, there is a distinct need for longitudinal research to evaluate the long-term effects of educational disruptions on nurses' professional adaptation more comprehensively. Such studies would not only validate our initial insights but also provide a more robust understanding of the impacts, enhancing the reliability and applicability of conclusions drawn from this research. Future studies should aim to employ varied study designs and broader sampling strategies to build on our findings and support the development of interventions to better prepare nursing professionals for unforeseen challenges.

## Figures and Tables

**Table 1:** Font Roja Questionnaire factors.

Factors	Definition
Satisfaction with the physical work environment	Grade of satisfaction with the physical and ergonomic characteristics of the workplace
Job monotony	Grade of routine of the relationships with colleagues, and grade of lack of variety in the work performed
Extrinsic status characteristics	Grade of work recognition in terms of salary, confidence, and independence
Interpersonal relationship with colleagues	Grade of satisfaction produced by relationships with colleagues
Interpersonal relationship with superiors	Grade of consciousness about what is expected of the worker by their superiors
Career advancement	Grade of ability to achieve professional promotion and recognition
Job pressure	Grade of overburden due to the work position
Professional competence	Grade of coincidence between professional training and work position
Job-related stress	Grade of stress induced by the profession of the worker and reflected by fatigue and the grade of responsibility perceived
Job satisfaction	Grade of satisfaction conditioned by the work position

Source adapted from Suárez et al. [20].

**Table 2:** Sociodemographic and academic characterization.

Variables	Frequencies *n* = 390 *n* (%) average (SD)				
Gender					
Female	318 (81.5%)				
Male	72 (18.5%)				
Age					
Range 20–42 years					
$\overset{¯}{X}$ (sd)	24.35 (2.876)				
*M*_*o*_	23				
Social status					
Single	358 (91.8%)				
Married	10 (2.6%)				
Another situation	22 (5.6%)				
Region where the degree is carried out					
Andalusia	141 (36.2%)				
Valencian community	58 (14.9%)				
Madrid	32 (8.2%)				
Catalonia	24 (6.2%)				
Murcia	19 (4.9%)				
Castile and León	19 (4.9%)				
Estremadura	17 (4.4%)				
Galicia	14 (3.6%)				
Castilla la Mancha	13 (3.3%)				
Canary Islands	13 (3.3%)				
Other	40 (10.3%)				
Practised during the pandemic					
Yes	308 (78.9%)				
No	82 (21.1%)				
Clinical practice lost due to COVID					
1 month or less	94 (24%)				
From 1 to 3 months	219 (56.2%)				
From 3 to 6 months	53 (13.6%)				
More than 6 months	24 (6.2%)				
Graduation year					
2020	77 (19.7%)				
2021	141 (36.2%)				
2022	172 (44.1%)				
Practices lost by graduation year (months)	<1	1–3	3–6	6>	Total
2020	3.3%	13.6%	2.1%	0.8%	19.7%
2021	5.6%	21.3%	7.4%	1.8%	36.2%
2022	15.1%	20.5%	4.9%	3.6%	44.1%
Employment situation					
Currently works as a nurse	327 (83.8%)				
Does not currently work as a nurse	63 (16.2%)				
Clinical practices where they currently work					
Yes	256 (65.6%)				
No	134 (34.4%)				

**Table 3:** Job satisfaction (26–130 scores) according to gender and academic variables (*n* = 390).

Variables	Categories	Job satisfaction Front Roja		Test (*p* value)
		$\overset{¯}{x}$	SD	
Gender	Male	90.53	13.35	*t* = 1.83 (*p*=0.778)
	Female	**87.37**	12.50	

Graduation year	2020	**85.48**	14.58	*Z* = 1.98 (*p*=0.140)
	2021	89.00	13.21	
	2022	88.19	11.22	

Practised during the pandemic	Yes	88.39	12.60	*t* = −1.288 (*p*=0.587)
	No	**86.31**	13.00	

Clinical practice lost due to COVID	1 month or less	87.80	12.49	*Z* = 0.595 (*p*=0.618)
	From 1 to 3 months	88.41	13.26	
	From 3 to 6 months	87.78	11.51	
	More than 6 months	**84.79**	12.70	

Clinical practices where they currently work	Yes	88.66	12.43	*t* = −1.53 (*p*=0.128)
	No	**86.59**	13.14	

⁣^*∗*^In bold are the lowest average values for each category.

**Table 4:** Distribution of lowest scoring factors in job satisfaction according to gender and academic variables (*n* = 390).

Variables	Categories	Job satisfaction Front Roja factors								
		Job pressure			Professional competence			Job-related stress		
		$\overset{¯}{x}$	SD	Test (*p* value)	$\overset{¯}{x}$	SD	Test (*p* value)	$\overset{¯}{x}$	SD	Test (*p* value)
Gender	Male	3.02	1.26	*t* = 2.03 (*p*=0.043)⁣^*∗*^	3.27	1.01	*t* = 2.54 (*p*=0.011)⁣^*∗*^	3.19	0.49	*t* = 1.18 (*p*=0.237)
	Female	2.71	1.13		2.95	0.94		3.11	0.52	

Graduation year	2020	2.77	1.17	*Z* = 2.876 (*p*=0.058)	2.93	0.99	*Z* = 5.89 (*p*=0.003)⁣^*∗*^	3.11	0.53	*Z* = 0.978 (*p*=0.377)
	2021	2.94	1.19		3.23⁣^*∗∗*^	0.97		3.07	0.54	
	2022	2.62	1.11		2.87	0.91		3.16	0.49	

Practised during the pandemic	Yes	2.78	1.18	*t* = −0.393 (*p*=0.695)	3.03	0.96	*t* = −0.712 (*p*=0.477)	3.13	0.51	*t* = −0.751 (*p*=0.453)
	No	2.72	1.11		2.95	0.95		3.09	0.53	

Clinical practice lost due to COVID	<1 mth	2.92	1.21	*Z* = 1.15 (*p*=0.329)	3.00	1.01	*Z* = 0.388 (*p*=0.762)	3.14	0.55	*Z* = 0.135 (*p*=0.939)
	1–3 mths	2.77	1.1		3.05	0.97		3.12	0.51	
	3–6 mths	2.65	0.97		2.93	0.89		3.11	0.51	
	6 mths <	2.50	1.01		2.88	0.86		3.06	0.55	

Clinical practices where they currently work	Yes	2.85	1.15	*t* = −1.77 (*p*=0.078)	3.03	0.98	*t* = −0.473 (*p*=0.636)	3.11	0.51	*t* = −0.501 (*p*=0.616)
	No	2.63	1.19		2.98	0.93		3.14	0.53	

⁣^*∗*^Statistically significant values (*p* < 0.05). ⁣^*∗∗*^The highest average in professional competence concerning the year of graduation revealed statistical differences solely between 2022 and 2021, supported by a Bonferroni test (*p*=0.003).

## Data Availability

The data used to support the findings of this study are available from the corresponding author upon request.
